# Reverse Abdominoplasty Flap in Reconstruction of Post-Bilateral Mastectomies Anterior Chest Wall Defect

**DOI:** 10.1155/2014/942078

**Published:** 2014-08-04

**Authors:** William HC Tiong, Normala Hj Basiron

**Affiliations:** Department of Plastic and Reconstructive Surgery, Hospital Kuala Lumpur, Jalan Pahang, 50586 Kuala Lumpur, Malaysia

## Abstract

Reverse abdominoplasty was originally described for epigastric lift. Since the work by Baroudi and Huger in the 1970s, it has become clear that reverse abdominoplasty application can be extended beyond just aesthetic procedure. Through the knowledge of anterior abdominal wall vascularity, its application had included reconstructive prospect in the coverage of various chest wall defects. To date, reverse abdominoplasty flap has been used to reconstruct unilateral anterior chest wall defect or for larger defect but only in combination with other reconstructive techniques. Here, we presented a case where it is used as a standalone flap to reconstruct bilateral anterior chest wall soft tissue defect post-bilateral mastectomies in oncological resection. In conclusion, reverse abdominoplasty flap provided us with a simple, faster, and satisfactory reconstructive outcome.

## 1. Introduction

Reverse abdominoplasty is traditionally used for correction of excess supraumbilical skin by performing epigastric lift through submammary incision [1]. This was first described by Rebello and Franco in 1972 [2]. Since the work of Baroudi et al. and Huger in the 1970s, the knowledge of vascular network of anterior abdominal wall has been well established and formed the basis of many cosmetic and reconstructive procedures [3, 4]. Further work by Lockwood expanded their use to include body contouring surgery [5, 6]. To date, there has been a paucity of literature on the use of reverse abdominoplasty flap for bilateral anterior chest wall soft tissue reconstruction after oncological resection [2]. Here, we presented our experience of using reverse abdominoplasty flap for bilateral anterior chest wall soft tissue reconstruction following bilateral mastectomies for a patient with bilateral breast cancer.

## 2. Case Report

**Figure 1:**
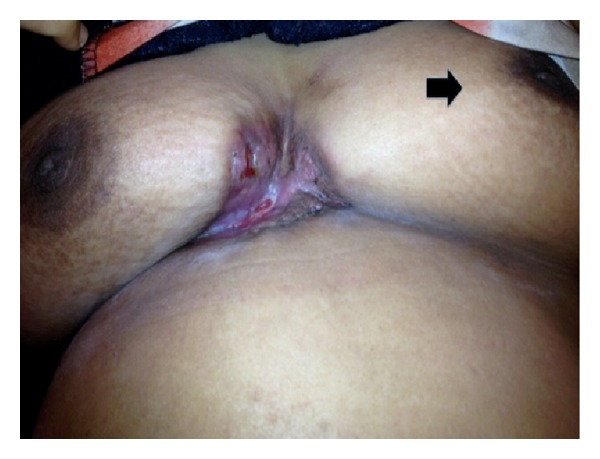
Infiltrating, ulcerative breast cancer (5 × 4 cm) from the inferomedial quadrant of the right breast encroaching to contralateral breast due to delayed presentation. Note that there was incidental finding of another breast lump (3 × 2 cm) over the central quadrant of the left breast as illustrated by the black arrow.

A forty-six-year-old healthy woman with 18 months history of ulcerating, fungative ulcer over the inferomedial quadrant of her right breast and a hard nodule over the central quadrant of her left breast was referred to our plastic and reconstructive surgery unit. Investigations had revealed that her breast cancer on the right side had infiltrated right pectoralis major muscle and encroached to the contralateral breast (Figure 1). She also had left axillary lymphadenopathy. She was scheduled for urgent bilateral mastectomies under breast surgical team and was referred for assessment of bilateral anterior chest wall reconstruction. Prior to this, she had received neoadjuvant radiotherapy for her locally advanced infiltrating ductal carcinoma (T_4_  N_1_  M_0_). She had, otherwise, an unremarkable past medical history.

**Figure 2:**
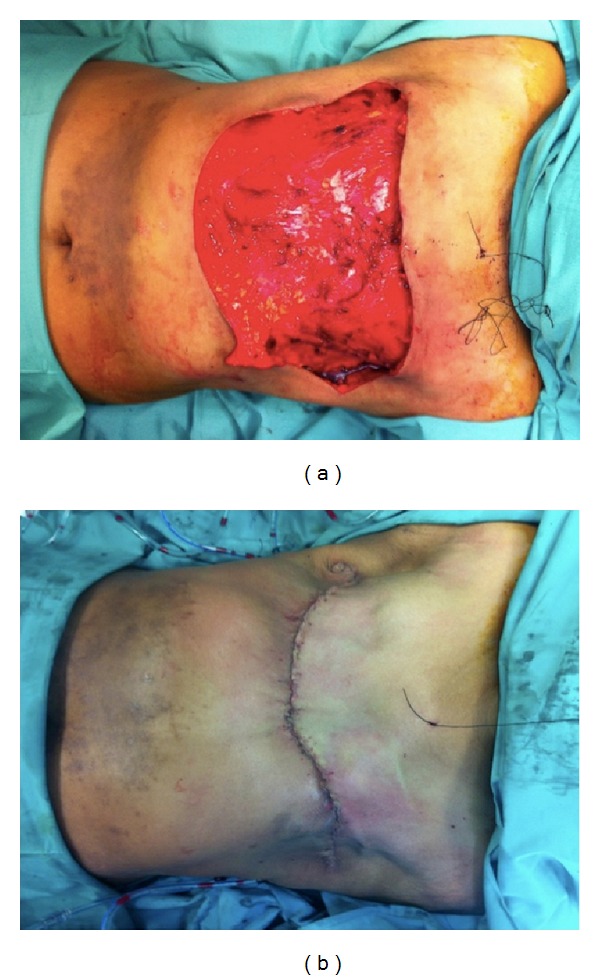
(a) Soft tissue defect over the anterior chest wall following bilateral mastectomies measuring 15 × 36 cm with exposed ribs and intercostal muscles on the right side and pectoralis major muscle on the left. Also of note is the skin discoloration following neoadjuvant radiotherapy. (b) Complete closure of anterior chest wall soft tissue defect following advancement of reverse abdominoplasty flap superiorly. Closure was done in layers with nonabsorbable sutures.

Intraoperatively, bilateral mastectomies including the right pectoralis major muscle were removed, and bilateral axillary clearance was carried out. The resulting soft tissue defect across her bilateral anterior chest wall was measured 15 × 36 cm with exposed ribs and intercostal muscles on the right side and pectoralis major muscle on the left (Figure 2(a)). Reverse abdominoplasty flap was raised superior to periumbilico-epigastric region to preserve the periumbilical perforators and advanced superiorly to close the defect.

**Figure 3:**
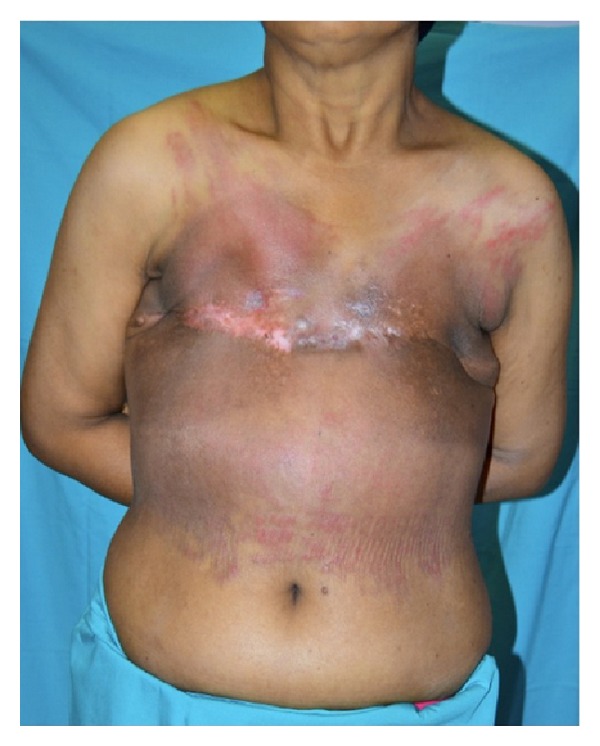
At three-month followup, the wound had fully settled with acceptable scar. The patient was offered breast reconstruction but declined.

Figure 2(b) showed complete closure of bilateral anterior chest wall soft tissue defect with reverse abdominoplasty flap. At two weeks following operation, there was minor chest wound dehiscence due to marginal skin flap edge necrosis. This was treated conservatively with dressing for 4 weeks with eventual, unremarkable wound healing (Figure 3). Currently, she remained disease-free and continued to decline breast reconstruction.

### 2.1. Surgical Technique

Following bilateral mastectomies and bilateral axillary clearance under the breast surgical team, the surgical field was resterilized and draped from the neck and shoulder down to suprapubic region with povidone-iodine. The inferior resection margin of the mastectomies constituted the superior border of the reverse abdominoplasty flap. The flap was elevated subfascially with its superficial fascial system (SFS) off the anterior chest wall fascia and fascia of Gallaudet of anterior abdominal wall. The flap was undermined caudally in a U-shaped distribution, stopping just 3 cm before the superior border of the umbilicus. This was necessary to avoid damaging the periumbilical perforators from the epigastric system. Care was taken to avoid excessive undermining laterally beyond the anterior axillary lines to prevent unnecessary devascularization of the flap from the intercostal and subcostal systems. Two suction drains were placed on each side, deep to the advanced flap and exteriorized laterally (Figure 2(a)). The drains were secured with 3/0 silk sutures. The flap was then advanced superiorly over the chest wall defect. It is important at this stage to avoid closure of the defect in excessive tension. The defect was closed in three layers. The SFS of the reverse abdominoplasty flap was secured to the SFS of the chest, pectoralis and intercostal fascia, and/or the periosteum of the ribs with 2/0 prolene sutures. Skin closure was performed with 3/0 prolene intradermally and running cutaneous 4/0 prolene sutures. Steri-Strips were applied across the wound and the wound was dressed with Surgical Aquacel.

## 3. Discussion

Breast cancer is the most common cancer to strike women and the second most common cancer in the world to kill women [7]. The overall incidence of bilateral breast cancer, as in our patient, ranges from 1.4 to as high as 12% [8]. Although the defect as a result of mastectomy is devastating both physically and mentally to the patient, there are numerous breast reconstructive options available today [9]. However, not all women prefer and opt for breast reconstruction and not all breast cancers are suitable for breast conserving surgery [9, 10]. As for the patient in our case, who has stage IIIB breast cancer, she opted for bilateral mastectomies with anterior chest wall reconstruction instead.

Chest wall defect can arise from its resection for a variety of conditions such as primary and secondary tumours of the chest wall or the sternum, lung cancer, infection, radionecrosis, or trauma [11]. The defect was classified by Whalen in 1953, as simple or compound defect based on the type of tissue loss [12]. The reconstructive options are myriad, dependent upon the size of the defects, the loss of structural support, the volume of dead space, and the vascularity of the wound [13, 14]. In our case, the component that required replacement was only the soft tissue. For midline sternal and anterolateral chest defects, various reconstructive methods have been described in the literature, ranging from direct closure of small defect to skin grafting to locoregional flaps and ultimately free flaps [13, 14]. The nonexhaustive list of flaps available for use included pectoralis major, latissimus dorsi, omentum, rectus abdominis, external oblique, serratus anterior, and the transverse thoracoabdominal flaps [13, 14].

Reverse abdominoplasty was originally described by Rebello and Franco in 1972 [2] It was a procedure advocated for aesthetic contouring of the upper anterior trunk and more recently employed in post-massive weight loss surgery [5]. Through improved anatomical understanding of anterior abdominal wall by the work of Lockwood and Huger, the application of reverse abdominoplasty has somewhat expanded from its original inception [4, 6].

The use of reverse abdominoplasty flap as a standalone reconstructive modality for chest wall soft tissue defect is uncommon and is often used in conjunction with other reconstructive techniques [2]. Its use has been either as a random or pedicled fasciocutaneous flap or as part of a musculocutaneous flap [2]. It has a robust blood supply and is a versatile and safe flap to use for reconstructive purposes [4]. Unlike others in the literature that described it for unilateral anterior chest wall soft tissue defect, we expanded its application to the reconstruction of bilateral anterior chest wall soft tissue defect secondary to bilateral mastectomies. It provided a good skin colour and texture match and lacked significant donor site morbidity. We felt that it was also easier and faster to perform with less cumbersome followup. It provided us with shorter operative time and postoperative recovery with less risk of complete flap failure even against the backdrop of radiotherapy.

## 4. Conclusion

Reverse abdominoplasty flap can in principle be considered as an alternative for bilateral anterior chest wall soft tissue reconstruction in patients who had bilateral mastectomies but declined breast reconstruction. It provides a mean by which we can offer such a patient a reconstruction that is robust and safe while providing simple soft tissue coverage of the defect.
